# Core Element Cloning, Cis-Element Mapping and Serum Regulation of the Human *EphB4* Promoter: A Novel TATA-Less Inr/MTE/DPE-Like Regulated Gene

**DOI:** 10.3390/genes10120997

**Published:** 2019-12-02

**Authors:** Pierluigi Scalia, Stephen J. Williams, Antonio Giordano

**Affiliations:** 1 Sbarro Institute for Cancer Research and Molecular Medicine, Biology Department, CST, Temple University, Philadelphia, PA 19122, USA; sjwilliamspa@comcast.net (S.J.W.); giordano@temple.edu (A.G.); 2 Istituto Somatogene per la Oncologia Personalizzata e la Ricerca Onco-Genomica, 93100 Caltanissetta, Italy; 3 Somatolink Foundation, Inc, Philadelphia, PA 19102, USA; 4 Dept of Medical Biotechnology, University of Siena, 53100 Siena, Italy

**Keywords:** polymerase-II, TATA(box)-less promoter, motif-of-ten element, downstream promoter element, DPE-like repeated motif, Sp1, HoxA9, insulin-like growth factor-II

## Abstract

The *EphB4* gene encodes for a transmembrane tyrosine kinase receptor involved in embryonic blood vessel differentiation and cancer development. Although EphB4 is known to be regulated at the post-translational level, little is known about its gene regulation. The present study describes the core promoter elements’ identification and cloning, the cis-regulatory elements’ mapping and the serum regulation of the human *EphB4* gene promoter region. Using bioinformatic analysis, Sanger sequencing and recombinant DNA technology, we analyzed the *EphB4* gene upstream region spanning +40/−1509 from the actual transcription start site (TSS) and proved it to be a TATA-less gene promoter with dispersed regulatory elements characterized by a novel motif-of-ten element (MTE) at positions +18/+28, and a DPE-like motif and a DPE-like-repeated motif (DRM) spanning nt +27/+30 and +32 +35, respectively. We also mapped both proximal (multiple Sp1) and distal (HoxA9) trans-activating/dispersed cis-acting transcription factor (TF)-binding elements on the region we studied and used a transient transfection reporter assay to characterize its regulation by serum and IGF-II using *EphB4* promoter deletion constructs with or without the identified new DNA-binding elements. Altogether, these findings shed new light on the human *EphB4* promoter structure and regulation, suggesting mechanistic features conserved among Pol-II TATA-less genes phylogenetically shared from *Drosophila* to Human genomes.

## 1. Introduction

EphB4 is a member of the Eph/Ephrin tyrosine receptor family involved in the formation of blood vessels during embryonal development [1]. It is typically expressed in venous endothelial cells and interacts at the extracellular level with its binding partner Ephrin B2 which is expressed in arterial endothelial cells in order to establish a forward and reverse signal conveying its pro-angiogenic and differentiative stimuli [2,3]. EphB4 has also been found ectopically expressed in a variety of solid cancers where it participates to a number of oncogenic effects including angiogenesis, invasion and metastasis [4,5,6]. Therefore, the study of the basic mechanisms driving EphB4’s expression under both physiological and pathological circumstances bear intrinsic scientific and medical relevance. In this context, it is worth noting the finding of a post-genetic mechanism allowing the tight control of post-transcriptionally expressed EphB4 protein levels via an autocrine signal specifically conveyed by cancer secreted IGF-II and targeting EphB4 via a newly phospho-degron domain, ultimately skipping transcriptional control to allow cellular hyperexpression [7]. Nonetheless, due to the growing inter-regulatory network observed among angiogenic factors (such as VEGF, IGF-II and HIF) in conditions such as hypoxia, the characterization of the human EphB4 gene promoter, can potentially answer biologically relevant problems while providing actionable new scientific knowledge. Indeed, the first attempt to characterize the human EphB4 gene promoter was made by Bruhl et al. [3]. That study provided evidence about the role of homeobox domain transcription factor A9 (HoxA9) as a critical factor for EphB4 transcriptional activation. However, the authors assumed that a TATA-box or a non-canonical form of it was present and sufficient to explain their results in spite of the fact that no core promoter element was identified nor included in the gene reporter study. The present work was meant to fill for the previous fragmentary knowledge on the *EphB4* gene’s regulatory region, seek unidentified promoter elements and provide a widely usable model on the structure, functional map and serum regulation of the *EphB4* human gene promoter.

## 2. Materials and Methods

### 2.1. Molecular Biology Reagents

Restriction enzymes and DNA-end modifying enzymes used for cloning were purchased from New England Biolabs (Ipswich, MA, USA). All other molecular biology reagents, unless specified, were purchased from Sigma (St. Louis, MO, USA). Synthetic oligos used for + 40/+10 core promoter element reconstitution and cloning were purchased from IDT (Coralville, IA, USA).

### 2.2. Human EphB4 Promoter Sequencing and Bioinformatic Analysis

A 1509 nt PCR fragment containing the genomic regions corresponding to +7/−1509 (numbering based upon nt validated distance from inr A+1 position) was sequenced using Sanger method from a pGL3-Luc reporter plasmid provided by C. Urbich (Univ. Frankfurt, Germany) using GL2primer2 and RVprimer3, respectively. Chromosomal consensus at the genomic DNA level, a transcription factor (TF) motif search and motif/domain comparisons were all performed using nBlast (NCBI).

### 2.3. Human EphB4 Core Promoter and Luc-Reporter Construct Cloning

**Figure 1:**
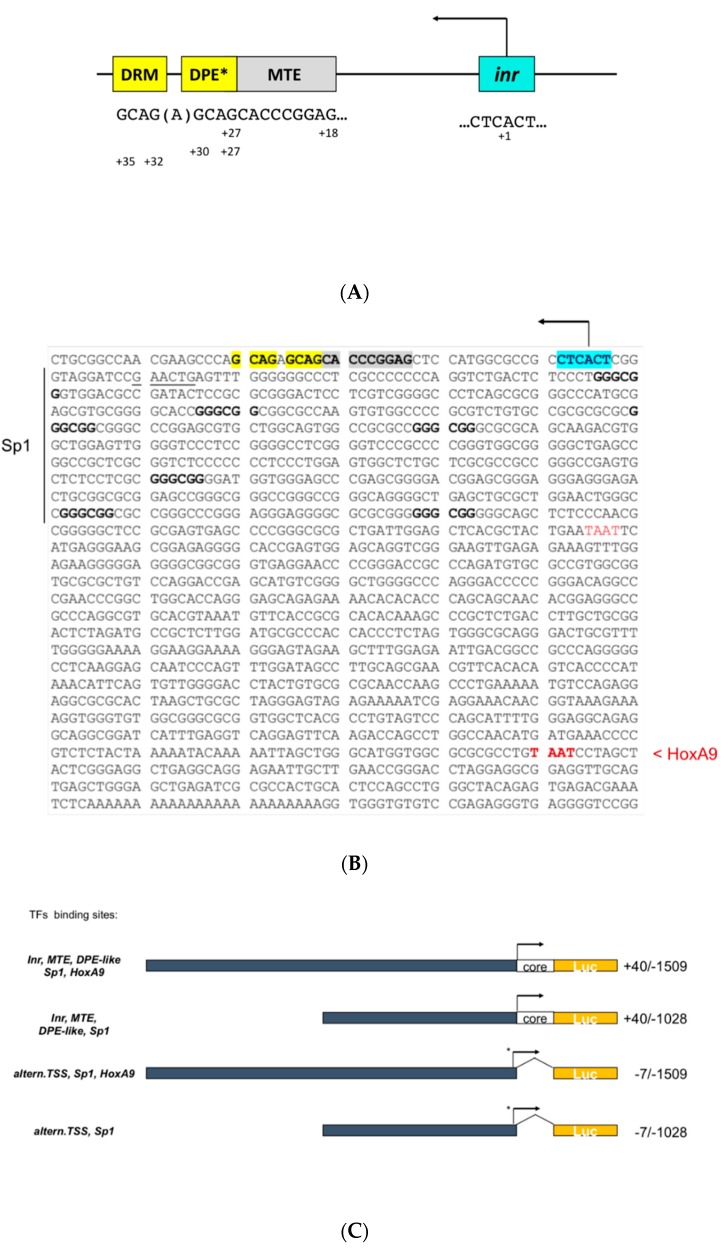
Core elements cloning and cis-element mapping of the Human *EphB4* promoter. (**A**) Graphic representation of *EphB4* core promoter elements with their motif sequences and positions compared to the transcription start site (TSS; A + 1) indicated. Asterisk: DPE-like motif (explained in text). (**B**) *EphB4* promoter sequence (+54/−1565 nt from TSS) with putative and/or functional transcription factor binding sites. Core elements: yellow, bold: DPE-like repeat motif; light gray: motif-of-ten element (MTE); light blue: initiator (Inr); underlined: putative alternative TSS; bold: multiple proximal Sp1 binding sites; red: putative or previously validated (red, bold) HoxA9 distal binding sites. (**C**) Human *EphB4* Luc-reporter constructs described in the study with the summary of the TF binding motifs present in each of the variants studied.

The +40/−1509 and +40/−1028 constructs were obtained by triple ligation reaction with sticky-ends fragments containing, respectively, the original −7 (BamHI)/1509 (or the −7/−1028 *EphB4*) promoter fragment(s) in a BamHI-KpnI fragment, and a KpnI-XhoI pGL3-luc plasmid backbone fragment along with a synthetic 54 nt synthetic reconstructed fragment spanning *EphB4* upstream promoter region from position +40 site from inr A+ position in a BamHI-XhoI frame. This last fragment was the result itself, of a double-synthetic, oligo pair (see sequence and cloning strategy in Figure S1) renaturation reaction (94 °C, 5 min, RT, 45 min) followed by gel purification of the final 54 nt product included in the triple ligation reaction. Ligation products were used for competent *Escherichia Coli* transformation JM109- Mix&Go^TM^, Zymo Research, Irvine, CA) and colony screening. Successful inclusion of the +40/–10 promoter region cloning was confirmed in the plasmid DNA extracted from selected positive colonies initially screened by NcoI double digest (since a NcoI RE site is present between Inr and motif-of-ten element (MTE)/DPE in the *EphB4* native core promoter sequence) followed by Sanger sequencing. As a result of the upstream cloning sequencing, two types of *EphB4* promoter constructs either containing the DPE or MTE cluster were generated, two of which ended at downstream position −1028 and two at position −1509, these last two containing a previously described *HoxA9*-binding region (see Figure 1C for the *EphB4* promoter constructs described in this study).

### 2.4. Cell Cultures, Transient Transfections and EphB4 Promoter Reporter Assay

MEFs with a null mutation for the IGF1R (R-cells) constitutively expressing either the human IGF1R (R+) or the insulin receptor isoform A (R-IR^A^) under puromycin-selective conditions were transiently transfected with the *EphB4* promoter constructs described herein. Following transfection in low serum optimized medium (GIBCO-Life Technologies, Carlsbad, CA, USA), cells were grown to sub-confluence and then serum starved for 16 h before stimulation by replacing the serum free media with either 10% containing media (DMEM, High glucose, 10%FBS, Sigma, St Louis, MO, USA) or serum free media containing 0.1% bovine serum albumin and 10 nM IGF-II (human recombinant, Sigma, St Louis, MO, USA) for following 16 h prior harvesting the cells in Luciferase assay buffer (Glo^TM^ Lysis buffer, Promega, Madison, WI, USA). The Luciferase reporter Dual-Glo^TM^ and Bright-Glo^TM^ kits and reagents used throughout the study were purchased by Promega. Results from luciferase assay are represented as means ± standard errors of the means (SEMs), and statistical analysis was by a Student’s *t*-test using unequal variance.

## 3. Results

### 3.1. Cloning of the Human EphB4 Core Promoter Reveals a TATA-less Promoter Regulated by Inr/MTE/DPE-Like Elements

In order to identify potential growth-factor-responsive elements in the human *EphB4* promoter, the sequence obtained from Sanger DNA sequencing of the genomic fragment spanning region −7/−1509 (gift of C.Urbich, U.Frankfurt) was used for a bioinformatic search for known transcription factors binding elements. This initial search did not result in the identification of canonical TATA box motifs spanning region −40 nt to +40 from the transcription start site (TSS), nor to the identification of the actual Inr sequence or other known core promoter elements within the previously cloned genomic fragments [3]. Nonetheless, this analysis allowed us to verify the presence of potential transcription factors binding motifs; namely, for Sp1 and HOXA9 (Figure 1B). In particular, our bioinformatic analysis in the original −7/−1509 cloned fragment revealed (a) seven canonical Sp1 DNA binding elements (GGGCGG) between −60 and −528 bp upstream to the putative TTS and two TAAT motifs at positions −598 and −1375, respectively, of which the most upstream (−1375) was previously shown to be a functional *homeobox A9* (*HoxA9*) regulatory motif whose mutation was able to ablate serum-induced transcription [3]. This finding suggested a mandatory role for HoxA9 in *EphB4* gene expression. However, in the absence of known core promoter elements within the previously cloned genomic region, we decided to investigate further. Therefore, we plotted the sequence spanning −7/−1509 in *EphB4*’s upstream flanking region against the chromosomal genomic context on chromosome 7 (GRCh37:7:100355490:100525847:1) obtained from the Ensemb database. Furthermore, in order to identify or rule out potential previously undetected core transcriptional elements, we restricted our bioinformatic search on the region comprised between the 5′ end of the *EphB4* transcribed gene and the transcription (A+1) start site which we identified in the previously invoked position (Figure 1). This analysis (Figure 1A,B) revealed indeed two core transcription regulatory motifs located within 40 nt downstream to the initiator (Inr) corresponding to an MTE motif spanning nt +18/+28 and a DPE-like motif spanning nt +27/+30 repeated at nt +32/+35 (that we called DPE-like-repeated motif, DRM). This finding, along with the absence of a TATA box in the previously cloned upstream region of the human *EphB4* promoter (Figure 1B) allowed us to redefine the human *EphB4* gene regulatory region as a TATA-less promoter with dispersed core motifs. The core promoter is the ultimate target of action of all the factors and coregulators that regulate the transcriptional activity of every gene controlled by RNA polymerase II (reviewed by Juven-Gershon and Kadonaga) [8]. Therefore, in order to study the effect of the newly identified *EphB4* core promoter elements on the overall transcriptional regulation of this gene, we decided to clone the missing +40/−6 regulatory sequence into two formerly described core promoter-deficient constructs (kind gift of C. Urbich) to reconstitute the regions spanning +40/−1509 and +40/−1028, respectively. The two novel Inr/MTE/DPE-like bearing constructs were cloned as described in Methods and Figure S1 and summarized in Figure 1C. As a result, two types of *EphB4* promoter constructs were generated either containing the Inr/MTE/DPE-like core elements or not, two of which ended at downstream position −1028 (containing multiple Sp1 motifs spanning −60/−528) and two at position −1509 (containing both multiple Sp1 and HoxA9-binding elements). A graphic summary of the *EphB4* promoter constructs and their known transcriptional regulatory elements is provided in Figure 1C.

### 3.2. The EphB4 Inr/MTE/DPE-Like Core Promoter

Since the MTE was first described in a number of *Drosophila* genes, we decided to perform a comparative/consensus analysis between the novel MTE sequence in the human EphB4 promoter and the previously reported *Drosophila* MTE sequences from Lim et al. [9], as shown in Table 1. In spite of the high level of similarity on the suggested consensus sequence, we found full divergence at positions +22 and +25, while we observed high or full consensus at positions +18 (100%), +19 (80%) +20 (100%), 27 (100%) and 28 (100%). Likely, the apparent divergence at key MTE conserved sites between human EphB4 and *Drosophila* MTE-bearing genes might be compensated by the synergism between adjacent MTE and DPE elements towards transcription initiation, as observed in other of Pol-II-regulated genes [8]. Theisen et al. [10] characterized three sub-regions of the MTE-DPE modular elements conferring particular advantages towards the interaction with TFIID, and in particular, with its subunits TAF9 and TAF6. These include sub-regions +17/+22, +27/+29 and +30/+33. Furthermore, Kadonaga [11] conveyed a summary on the DPE motif pointing at positions +24 and +28/+32 in Inr-containing core promoters as relevant towards this element capability to establish cooperative physical interaction with Pol-II-complex TFIID. Our analysis of the *EphB4* MTE/DPE motifs in the EphB4 promoter with the suggested consensus sequences of Thiesen et al. [10] and Kadonaga [11] to exert most efficient TFIID binding is consistent with an MTE motif with highest consensus in two sub-regions indicated at positions +17 and +25 and a DPE-like motif with a C instead of a G in position +24 and a 2 nt shift over the classically described positions at +29/+32 for the GACG core DPE motif, which we found at positions +27/+30 in the *EphB4* downstream promoter along with a full shared consensus (A) in position +28. Of interest in the *EphB4*, DPE-like downstream region is the presence of a DPE-like repeated GACG motif (DRM) at position +32/+35. It will be useful to determine the physical interaction features with the Pol-II core transcriptional machinery conferred by this new type of core promoter element configuration.

### 3.3. The Serum-Dependent Transcriptional Response of EphB4 TATA-Less Inr/MTE/DPE-Like Downstream Promoter Is Enhanced by Multiple Proximal Sp1 Consensus Motifs

**Figure 2:**
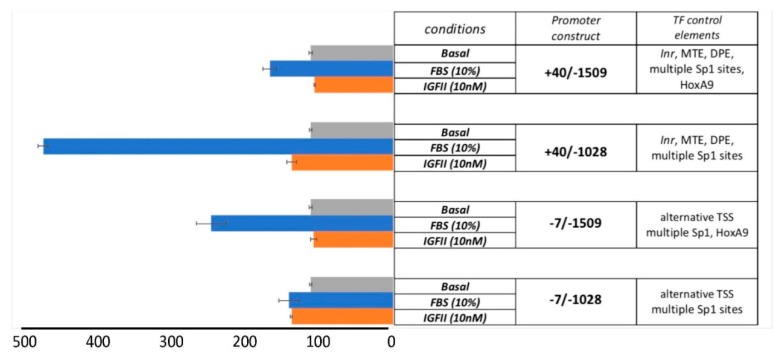
The effect of core promoter elements and cis-acting regulatory elements on serum-induced *EphB4* gene transcription in R-IRA cells. Luciferase assay in R-IR^A^ MEF cells transiently transfected with the described hEphB4 promoter reporter constructs.

Mouse embryo fibroblast cells we previously generated [7,12] were used to study the *EphB4* promoter transcriptional activation by serum, as described under methods. As summarized in Figure 2, we observed the highest stimulatory effect (4.22 fold over unstimulated basal; *p* ≤ 0.0001) with the *EphB4* promoter construct +40/−1028 bearing the identified Inr/MTE/DPE-like downstream elements and the defect of the region bearing the *HoxA9* transactivation motif at −1375. This result suggests an underscored role for the multiple proximal *Sp1*-binding elements in driving *EphB4* serum-stimulated transcription. This result is also consistent with the well-known synergistic ability of multiple Sp1 complexes to induce the transcription of TATA-less genes due to the efficient recruitment of TAF4 and TAF7 (part of TF_II_D) towards transcription initiation, an effect known as Sp1 super-activation (reviewed by Wierstra [13]). The serum response in +40/−1509, in which both non-core cis-transactivating elements (multiple *Sp1*+*HoxA9*) are present, provided only a discrete activation suggesting a competitive or negative cooperative role of Sp1 and HoxA9 (compare +40/−1509 with +40/−1028). When looking at the serum-response observed with the *EphB4* core-promoter (Inr/MTE/DPE-like)-deficient constructs (−7/−1509 and −7/−1028), our results are consistent with the results previously obtained by Bruhl et al. [3] with the *HoxA9* distant motif showing the most effective transactivation activity towards serum-stimulated *EphB4* transcription. On the other hand, the isolated proximal Sp1-binding elements-spanning region −60/−528 (contained in the −7/−1028 construct) did not appear to play a major role in *EphB4* serum-dependent transcription in absence of the Inr/MTE/DPE-like core elements. To explain the transcriptional activity that both we (Figure 2) and Bruhl et al. [3] observed with the −7/1509 and −7/1028 constructs, we postulated the presence of a subsidiary non-canonical start site that upon search for previously described alternative consensus TSS sequences [14] could be provided by the motif GTCAAG (5′- > 3′) at position −20 to −15 from Inr_+A_. Although such hypothesis remains to be confirmed, it provides a likely explanation for the basic and HoxA9-enhanced transcriptional activity observed in absence of the Inr/MTE/DPE-like elements (−7/−1509 and −7/−1028). Under the studied conditions, IGF-II stimulation (10nM) did not exert transcriptional effects (not significant at *p* > 0.6 for both constructs) similar to those observed with serum, although a minimum effect was observed in *Sp1*+/*HoxA9* motif-less promoters, irrespective of the presence of the Inr /MTE/DPE-like downstream elements (+40/−1028 and −7/1028) (Figure 2).

## 4. Discussion

The core promoter in human genes is the region from −40 to +40 and flanks the transcription start site (TSS) at A+1 [15]. There are no universal core promoter elements that are present in all promoters. Different types of core promoters are transcribed by different sets of transcription factors and exhibit distinct properties, such as specific interactions with transcriptional enhancers, that are determined by the presence or absence of particular core promoter motifs [15]. Inr is the most common core promoter element in humans. In fact, it has been estimated that about 50% of human core promoters contain an Inr [16]. Although the Inr is enriched at promoters with focused transcriptional start sites, it is also found randomly distributed throughout the genome. Therefore, a consensus Inr alone does not constitute a promoter. On the same token, it has been estimated that <25% of human core promoters contain the well-known TATA box or a TATA-like sequence [16,17,18]. The MTE (motif-of-ten-element) is a phylogenetically conserved Pol-II core promoter element located precisely at positions +18 to +27 relative to A+1 in the initiator (Inr) element [9]. DPE is a TAF recognition site (for TAF_II_60/40 subcomplex or as part of TFIID) located about 30 nucleotides downstream of the transcription start site of many TATA-box-deficient (TATA-less) promoters from *Drosophila* and humans [19]. In *Drosophila*, about 26% of promoters are TATA-less and bear a DPE motif [20]. Furthermore, MTE and DPE are often found associated in TATA-less promoters and synergize with Inr towards pre-initiation complex formation [18]. Although the MTE functions independently of the TATA-box and DPE, it exhibits strong synergism in their presence [9]. Theisen et al. [10] found that MTE promotes the binding of purified TFIID to the core promoter. Their study further describes potential interaction of TAF6 and TAF9 subunits of TFIID with the MTE-DPE core module at three sub-regions located at +17/+22, +27/+29 and +30/+33. Our finding of a partial consensus on two of these three sub-regions by the *EphB4* MTE-DPE-like module at these positions might support a scenario with similar core promoter interactions. However, the DPE-like repeat (GACG) we found in the *EphB4* downstream promoter at positions +27/+30 and +32/+35 (herein called DRM for DPE-like repeated motif), separated by one “A” nt bridge in position +31, is in apparent contrast with the classic DPE motif spanning positions +28/+32 [11]. This finding, we believe, may offer a potential model to understand the function of this Inr/MTE-DPE-like modular element both for *EphB4* gene regulation and for other TATA-less genes bearing similar downstream core elements. We recently found IGF-II to be a key driver of protein EphB4 ectopic expression in malignant mesothelioma cells and we identified a ubiquitin/UPS-associated post-transcriptional mechanism tightly regulating EphB4 protein degradation/stabilization [7]. The lack of significant transcriptional stimulation of the *EphB4* promoter by IGF-II compared to serum in R-IR^A^ cells would therefore suggest that IGF-II is not a major driver of the *EphB4* gene transcriptional activation and supports our previous finding that the strong dependence of EphB4 protein expression by IGF-II is linked to a post-transcriptional mechanism. Indeed, the conclusion in which we claim that IGF-II is not a major driver of *EphB4* transcriptional activation is based upon the fact that IGF-II is a major serum component. Therefore, the difference observed in transcriptional activation between serum and IGF-II on the studied regulatory promoter regions is strong evidence that other growth factors than IGF-II must be responsible for the serum effects observed under the conditions tested. Nonetheless, we cannot rule out an IGF-II specific role in the *EphB4* gene’s transcriptional regulation via far distant elements and/or under different contextual cellular events (e.g., hypoxia) not tested in our present study.

In summary, using DNA sequencing, recombinant DNA cloning, bioinformatics and a gene reporter assay, here, we reported the human *EphB4* gene promoter to be a serum-responsive TATA-less Inr/MTE/DPE-like-driven promoter. Our findings support a synergistic role of the multiple Sp1 motif containing proximal region with the identified Inr/MTE/DPE-like core promoter elements for exerting a full serum transcriptional response. Furthermore, our study shows that serum-dependent *EphB4* transcriptional regulation is affected by a negative cooperative effect between proximal and distal regulatory elements; namely, between the upstream proximal promoter region containing multiple Sp1-binding motifs (−60/−528) and the distant promoter region bearing a HoxA9-binding motif (at −1375), respectively. Further studies will need to clarify the mechanistic and regulatory aspects linked to the identified *EphB4* downstream promoter elements and the competing contextual roles of this promoter cis-acting elements at the proximal and distal sites.

## Figures and Tables

**Table 1:** *hEphB4* MTE core promoter elements and consensus with *Drosophila* genes. The MTE consensus of human *EphB4* was compared to the consensus sequence obtained by other MTE core promoter elements from *Drosophila* genes previously reviewed by Lim et al. [9]. Explanation provided in the text.

Nt from TSS	+18	+19	+20	+21	+22	+23	+24	+25	+26	+27	+28	+29
*MTE consensus*	C	S	A	R	C	S	S	A	A	C	G	S
***H EphB4***	**C**	G	**A**	G	G	C	C	C	**A**	**C**	**G**	A
*CG4427*	C	G	A	A	C	G	C	A	A	C	G	G
*CG15312*	C	G	A	A	C	G	C	A	A	C	G	G
*CG14720*	C	G	A	A	C	C	A	A	A	C	G	G
*Hedgehog*	C	G	A	G	C	G	C	A	A	C	G	G
*CG15695*	C	G	A	G	C	G	G	A	G	C	G	G
*CG10479*	C	G	A	A	C	C	G	A	T	C	G	C
*Tollo*	C	G	A	G	C	C	G	A	G	C	G	G
*Side*	C	A	A	G	C	C	A	A	G	C	G	C
*CG12537*	C	A	A	G	C	G	G	A	G	C	G	G
***% consensus***	***100%***	***80%***	***100%***	***60%***	***0***	***50%***	***40%***	***0***	***50%***	***100%***	***100%***	***0***

## Supplementary Materials

The following are available online at https://www.mdpi.com/2073-4425/10/12/997/s1, Figure S1: Cloning strategy of human *EphB4* core promoter elements.
