# Supplementary material for: Core Element Cloning, Cis-Element Mapping and Serum Regulation of the Human EphB4 Promoter: A Novel TATA-Less Inr/MTE/DPE-Like Regulated Gene
Source: Genes (Basel). 2019 Dec 2;10(12):997. doi: 10.3390/genes10120997 (PMC6947382; doi:10.3390/genes10120997)
Supplement: Supplementary file 1 [file genes-10-00997-s001.pdf]

Suppl.Figure 1

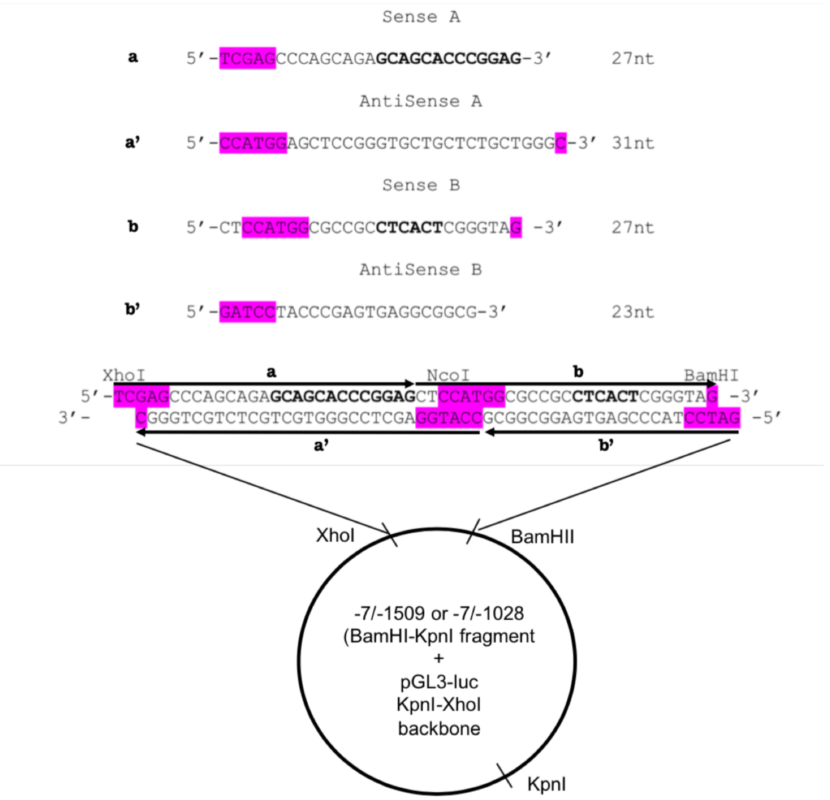

Supplementary Figure 1. Cloning strategy of human EphB4 core promoter elements. . “Sticky ends” partially complementary synthetic oligo pairs (a/a’ and b/b’) with sequence spanning the region corresponding to the nt +40/-10 of the human EphB4 promoter were used for double semi-complementary annealing by temperature denaturation/renaturation followed by a triple fragments ligation to reconstitute the EphB4 genomic sequence conveyed in the Luc-reporter constructs +40/-1028 and +40/-1509 used in this study.
